# Does the susceptibility vessel sign influence the effectiveness of intravenous thrombolysis before endovascular thrombectomy in acute ischaemic stroke?

**DOI:** 10.1093/esj/aakaf003

**Published:** 2026-01-01

**Authors:** Timothée Werlé, Florent Wijanto, Emilien Micard, Bailiang Chen, Marine Beaumont, Kevin Janot, Marco Pasi, Joseph Benzakoun, Jean Philippe Cottier, Bertrand Lapergue, Grégoire Boulouis, Fouzi Bala

**Affiliations:** Diagnostic and Interventional Neuroradiology Department, University Hospital of Tours, Tours, France; Department of Neurology, Foch Hospital, Versailles Saint-Quentin en Yvelines University, Suresnes, France; CIC, Innovation Technologique, CHRU-Nancy, INSERM, Université de Lorraine, Nancy, France; CIC, Innovation Technologique, CHRU-Nancy, INSERM, Université de Lorraine, Nancy, France; CIC, Innovation Technologique, CHRU-Nancy, INSERM, Université de Lorraine, Nancy, France; Diagnostic and Interventional Neuroradiology Department, University Hospital of Tours, Tours, France; Imaging Brain & Neuropsychiatry, (iBraiN) U1253, INSERM, Université de Tours, Tours, France; Department of Neurology, University Hospital of Tours INSERM, U1253, Tours, France; Neuroradiology Department, Hôpital Sainte Anne, GHU-Paris Psychiatrie et Neurosciences, Paris, France; Diagnostic and Interventional Neuroradiology Department, University Hospital of Tours, Tours, France; Imaging Brain & Neuropsychiatry, (iBraiN) U1253, INSERM, Université de Tours, Tours, France; Department of Neurology, Foch Hospital, Versailles Saint-Quentin en Yvelines University, Suresnes, France; Diagnostic and Interventional Neuroradiology Department, University Hospital of Tours, Tours, France; Imaging Brain & Neuropsychiatry, (iBraiN) U1253, INSERM, Université de Tours, Tours, France; Diagnostic and Interventional Neuroradiology Department, University Hospital of Tours, Tours, France; Imaging Brain & Neuropsychiatry, (iBraiN) U1253, INSERM, Université de Tours, Tours, France

**Keywords:** stroke, thrombolysis, thrombectomy, magnetic resonance imaging, occlusion

## Abstract

**Introduction:**

The benefit of intravenous thrombolysis (IVT) prior to EVT in acute ischaemic stroke (AIS) remains debated. We evaluated the association of the susceptibility vessel sign (SVS) with clinical and angiographic outcomes and assessed whether its presence modified the effect of IVT.

**Patients and methods:**

We retrospectively analysed patients with anterior circulation large vessel occlusion from the multicentre ETIS registry who underwent EVT. Susceptibility vessel sign presence and extent were assessed on MRI and categorised as binary (SVS− vs SVS+) and 3-class (SVS−, SVS+, SVS++) variables. Multivariable regression was used to evaluate associations and interactions between SVS and IVT for the primary (90-day mRS 0–2) and secondary (90-day ordinal mRS and mortality, first-pass expanded thrombolysis in cerebral infarction [eTICI] 2c-3 and final eTICI 2b-3) outcomes.

**Results:**

Among the 1250 patients analysed, 909 were included. Susceptibility vessel sign was present in 84.5% of patients and associated with improved 90-day mRS 0–2: adjusted odds ratio (aOR) 2.03; 95% CI, 1.18–3.46. No interaction between SVS and IVT was observed for clinical outcomes. However, SVS modified the effect of IVT on final TICI 2b-3 (*P*_interaction_ = .03): IVT + EVT was associated with higher odds of successful reperfusion in SVS+ patients (aOR 2.00; 95% CI, 1.28–3.52) but not in SVS− patients (aOR 0.60; 95% CI, 0.16–1.97). In a secondary analysis using 3-class SVS, only SVS++ (larger hyposignal) was significantly associated with better outcomes and showed interaction with IVT for final eTICI 2b-3.

**Conclusion:**

Susceptibility vessel sign, particularly SVS++, was associated with improved clinical outcomes and enhanced the effect of IVT on reperfusion success in EVT-treated AIS.

## Graphical Abstract

**Graphical Abstract:**
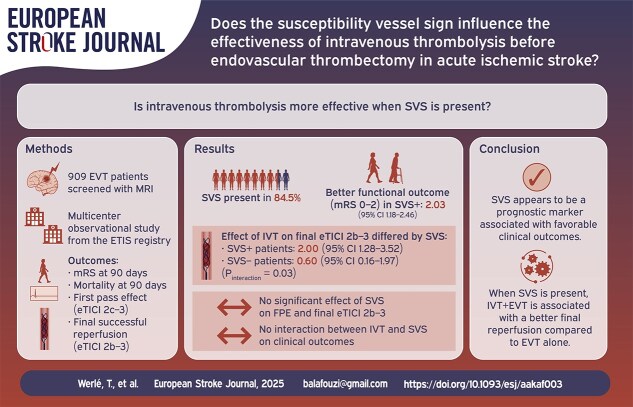

## Introduction

Combined intravenous thrombolysis (IVT) and EVT is the standard treatment of acute ischaemic stroke (AIS) with large vessel occlusion of the anterior circulation. The improving reperfusion strategies in ischaemic stroke individual participant data meta-analysis^1^ failed to demonstrate the non-inferiority of EVT alone relative to combined IVT and EVT. In this meta-analysis, no treatment effect heterogeneity was observed for prespecified subgroups except for time from onset to randomisation favouring thrombolysis administration in patients presenting earlier.

Thrombi responsible for ischaemic strokes exhibit variable composition, with some more responsive to IVT than others. Specifically, it has been shown that thrombi rich in red blood cells are more likely to be dissolved by thrombolysis than those rich in platelets and fibrin.^2^ Additional studies have also demonstrated that red blood cell-rich thrombi are associated with susceptibility vessel sign (SVS) artefacts on MRI using T2^*^-weighted gradient echo (GRE) or SWI.^3–5^ Based on these insights, identification of patient subgroups based on thrombus MRI signal may be useful in determining which patients would benefit or not from IVT added to EVT.

This study aims to evaluate the association of SVS with outcomes and to assess whether the presence or absence of SVS modifies the treatment effect of IVT plus EVT vs EVT alone, using data from a multicentre real-world stroke registry. We hypothesise that patients with SVS would respond better to added thrombolytic therapy and, therefore, would experience better outcomes.

## Patients and methods

### Study population

**Figure 1:**
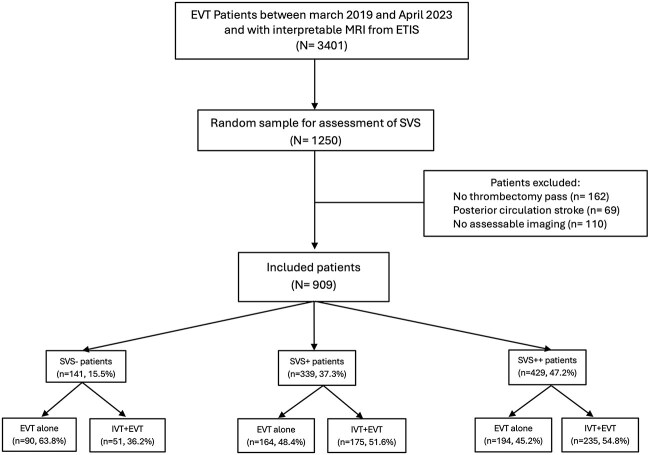
Study flow chart.

This study is a retrospective analysis of data from the Endovascular Treatment in Ischemic Stroke Registry (ETIS, ClinicalTrials.gov: NCT03776877), which is an ongoing multicentre prospective registry of 30 comprehensive stroke centres in France including adult patients with AIS due to an anterior or posterior circulation large vessel occlusion treated with EVT. Patients treated with EVT between March 2019 and April 2023 and with available and interpretable MRI images were considered for inclusion (*n* = 3401), see Figure 1 for the study flowchart. Study data are available from the corresponding author upon reasonable request.

Susceptibility vessel sign is not routinely adjudicated in ETIS. Because SVS reading for all patients in the registry was not feasible, a random sample of 1250 patients was selected for this study to minimise bias. After sampling, we excluded patients who did not undergo an intracranial thrombectomy pass (*n* = 162), patients with posterior circulation stroke (*n* = 69) and images that were not assessable at the level of occlusion due to motion or metallic artefacts (*n* = 110). Excluded patients had less severe strokes, longer onset to arterial access times and higher proportions of smoking (Table S1).

Our study was approved by research ethics boards at each participating centre and written informed consent was waived because of the retrospective design. This study adheres to the Strengthening the Reporting of Observational Studies in Epidemiology (STROBE) guidelines.^6^

### Imaging analysis

The presence or absence of SVS was assessed by 2 readers: a neuroradiology resident (T.W.) and an interventional neuroradiology consultant (F.B.) with 4 and 10 years of experience in stroke imaging interpretation, respectively. The cases were divided between the 2 readers, who were blinded to all clinical data except for the side of the stroke.

**Figure 2:**
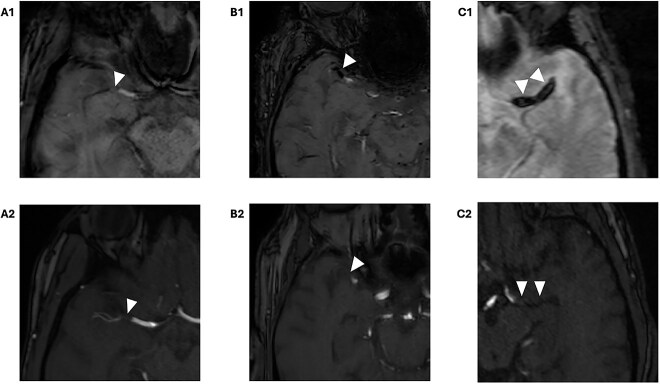
MRI examples of thrombus appearance, illustrating the different types based on the SVS status. (A) Right M1-MCA occlusion without SVS (arrowhead), A1 = SWI, A2 = 3D TOF. (B) Right M1-MCA occlusion with SVS+ (hyposignal similar to the non-occluded M1 segment, arrowhead), B1 = SWI, B2 = 3D TOF. (C) Left M1-MCA occlusion with SVS++ (hyposignal larger than the non-occluded M1 segment, arrowhead), C1 = T2-GRE, C2 = 3D TOF. Abbreviations: SVS = susceptibility vessel sign; SWI = susceptibility weighted imaging; TOF = time of flight; MCA = middle cerebral artery; GRE = gradient echo.

Susceptibility vessel sign was defined as a hypointense signal on GRE or SWI in the occluded vessel (Figure 2). If SVS was seen, we further distinguished 2 SVS subgroups based on the diameter of the hypointense signal: the SVS+ subgroup if the diameter of the hyposignal was visually similar to that of the contralateral artery and the SVS++ subgroup if the diameter of the hyposignal was visually larger than that of the contralateral artery (Figure 2). We conducted 2 analyses using different SVS variables defined as follows: (1) binary SVS (negative or positive SVS) and (2) 3-class SVS (SVS−, SVS+ and SVS++). We used these 2 different definitions as previous studies showed that thrombus composition and origin vary according to the size of its signal.^7^^,^^8^ Susceptibility vessel sign interrater agreement was calculated in a random 50 cases for both variables using κ statistics.

### Study outcomes

Clinical outcomes were mRS score 0–2, ordinal mRS and mortality at 90 days and SICH. mRS score was scored by board-certified stroke neurologists or trained research nurses blinded to imaging and clinical information at each participating centre. SICH was defined according to the European Cooperative Acute Stroke Study III criteria.^9^ Angiographic outcomes were first-pass effect (expanded thrombolysis in cerebral infarction [eTICI] 2c-3 after the first pass) and final successful reperfusion (eTICI 2b-3). Angiographic outcomes were adjudicated prospectively at each participating site by a neuroradiologist with at least 5 years’ experience. For this study, the primary outcome was 90-day mRS 0–2; all other outcomes were considered secondary.

### Statistical analysis

The primary analysis consisted of studying the association of the binary SVS variable (absent vs present) with outcomes and its interaction with IVT. Baseline clinical and imaging characteristics were compared between negative SVS and positive SVS patients using descriptive statistics. Quantitative variables were expressed as median (IQR) and categorical variables as numbers and proportions. The Wilcoxon rank-sum test was used for continuous variables and the χ-square test for categorical data. Unadjusted and adjusted binary logistic and ordinal regressions were performed to assess the association between SVS status and the study outcomes. For clinical outcomes, adjustments were made for age, sex, baseline NIHSS, history of stroke, history of atrial fibrillation, time from onset to arterial access, Alberta Stroke Program Early CT Score, IVT and type of MRI sequence (T2^*^-GRE vs SWI). For angiographic outcomes, we adjusted for age, occlusion location, IVT, history of stroke, history of atrial fibrillation, onset to arterial access time, first EVT strategy (aspiration alone vs stent retriever alone or combined approach) and MRI sequence. These confounders were selected based on the literature and baseline differences between EVT alone and IVT-EVT groups in the univariable analyses. To assess the effect modification of IVT by SVS status for each outcome, multiplicative interaction terms (“intravenous thrombolysis” X “SVS status”) were added to the adjusted models.

In the secondary analysis, we used 3-class SVS (SVS−, SVS + and SVS++) as a categorical and ordinal variable and repeated the above analyses to assess whether the difference in degree of thrombus hyposignal is clinically relevant.

Effect size estimates were reported as adjusted odds ratios (aORs) for binary outcomes or adjusted common odds ratios (acORs) for a worse outcome for ordinal mRS with 95% CIs. Statistical significance was defined as 2-tailed *P* < .05. All analyses were conducted using Stata/MP 17.0 (STATA LLC, Corp.).

## Results

Among the sample of 1250 patients from the ETIS registry, 909 patients met the inclusion criteria of this study. Median (IQR) age was 73 years (60–82) and 482 (50.8%) were female, 141 (15.5%) were SVS-negative and 768 (84.5%) were SVS-positive. Baseline characteristics stratified by SVS status and the comparison between EVT alone and IVT + EVT subgroups are summarised in Table 1 and Table S2, respectively. Patients with positive SVS were older (73 [62–82] years vs 68 [57–80] years, *P* = .02), and more commonly received IVT before EVT (410 [53.4%] vs 52 [36.2%], *P* < .001). Alteplase was the main thrombolytic agent used and tenecteplase was only used in 66 (7%) patients. The prevalence of SVS was higher on SWI (91.3%) vs T2^*^-GRE sequences (77.1%), *P* < .001; see Table S3 for baseline characteristics by sequence type. Interrater agreement was excellent for both binary SVS (κ 0.87; 95% CI, 0.71–1.00) and 3-class SVS (κ 0.81; 95% CI, 0.68–0.90).

**Table 1:** Baseline characteristics of included patients stratified by binary SVS status (*n* = 909).

	**SVS negative (*n* = 141)**	**SVS positive (*n* = 768)**	***P* value**
Age, median (IQR)	68 (57–80)	73 (62–82)	**.02**
Female sex	62 (44.0)	385 (50.1)	.18
Medical history			
Hypertension	72 (54.1)	429 (57.1)	.53
Diabetes	27 (20.6)	109 (14.6)	.08
Dyslipidemia	41 (31.3)	220 (29.6)	.70
Atrial fibrillation	21 (15.8)	151 (20.6)	.20
Previous stroke	22 (16.4)	94 (12.6)	.23
Smoking	27 (20.8)	110 (15.6)	.14
Prestroke mRS 0–2	124 (92.0)	669 (95.0)	.14
Antithrombotic medication	55 (40.4)	278 (37.7)	.55
IV thrombolysis	51 (36.2)	410 (53.4)	**<.001**
Baseline NIHSS, median (IQR)	15 (9–19)	16 (10–20)	.21
Onset to arterial access time	265 (180–351)	253 (190–326)	.62
Stroke aetiology			.31
Atherosclerosis	21 (16.4)	97 (13.5)	
Cardioembolic	45 (35.2)	303 (42.1)	
Other	62 (48.4)	319 (44.4)	
Imaging characteristics			
MRI sequence^a^			**<.001**
T2^*^-GRE	100 (70.9)	338 (44.0)	
SWI	41 (29.1)	430 (56.0)	
Occlusion location			.38
ICA	27 (19.2)	181 (23.8)	
M1-MCA	83 (58.9)	433 (57.0)	
M2-MCA	27 (19.2)	114 (15.0)	
Other/Distal occlusions	4 (2.9)	32 (4.2)	
Baseline ASPECTS (IQR)	8 (5–9)	8 (6–9)	.97
First EVT strategy			.85
Contact aspiration alone	55 (41.3)	313 (42.2)	
Stent retriever alone or combined with aspiration	78 (58.6)	428 (57.7)	

^a^Numbers in brackets are row-wise proportions. Values in bold indicate statistical significance.

Abbreviations: ASPECTS = Alberta Stroke Program Early CT Score; EVT = endovascular thrombectomy; GRE = gradient echo; ICA = internal carotid artery; IQR = interquartile range; IV = intravenous; MCA = middle cerebral artery; MRI = magnetic resonance imaging; NIHSS = National Institutes of Health Stroke Score; SVS = susceptibility vessel sign; SWI = susceptibility weighted imaging.

### Primary analysis using binary SVS variable

The presence of SVS was associated with higher odds of 90-day mRS 0–2 (aOR = 2.00; 95% CI, 1.18–3.46), lower 90-day ordinal mRS (acOR 0.53; 95% CI, 0.35–0.80) and lower mortality (aOR 0.44; 95% CI, 0.25–0.77), but not with SICH in the unadjusted and adjusted analyses (Table 2). No interaction between SVS and IVT was found for all clinical outcomes (Table 3).

**Table 2:** Association of binary SVS with clinical and angiographic outcomes.

	**SVS negative (*n* = 141)**	**SVS positive (*n* = 768)**	***P* value**	**Unadj OR**	**Adj OR**
90-day mRS 0–2	41 (31.8)	317 (44.5)	.01	1.17 (1.15–2.56)	2.03 (1.18–3.46)
90-day mRS (median, IQR)	4 (2–6)	3 (1–5)	.01	0.57 (0.41–0.79)	0.53 (0.35–0.80)
90-day death	35 (27.1)	128 (17.9)	.02	0.59 (0.38–0.90)	0.44 (0.25–0.77)
SICH^a^	10 (7.9)	56 (8.6)	.80	1.09 (0.54–2.20)	0.88 (0.39–1.96)
FPE	47 (37.9)	276 (39.9)	.68	1.09 (0.73–1.61)	0.97 (0.62–1.51)
Final eTICI 2b-3	116 (84.7)	670 (90.0)	.15	1.46 (0.87–2.45)	1.29 (0.70–2.36)

^a^Data were missing in 133 patients.

Abbreviations: Adj = adjusted; CI = confidence interval; d = days; eTICI = expanded treatment in cerebral infarction; mRS = modified Rankin Scale; FPE = first-pass effect; OR = odds ratio; SVS = susceptibility vessel sign; Unadj = unadjusted; SICH = symptomatic intracranial haemorrhage.

**Table 3:** Treatment effect of combined IVT and EVT vs EVT alone for the study outcomes stratified by binary SVS status.

	**EVT alone (*n*/*N* [%])**	**IVT + EVT (*n*/*N* [%])**	**Adj OR**	***P***_**interaction**_
90-day mRS 0–2				.40
Negative SVS	21/83 (25.3)	20/46 (43.5)	3.52 (0.98–12.54)	
Positive SVS	126/333 (37.8)	191/380 (50.3)	1.50 (1.02–2.21)	
90-day mRS (median [IQR])				.81
Negative SVS	4 (2–6)	3 (1–6)	0.56 (0.24–1.27)	
Positive SVS	3 (1–5)	2 (1–4)	0.74 (0.54–1.00)	
90-day mortality				.44
Negative SVS	21/83 (25.3)	14/46 (30.4)	0.98 (0.33–2.93)	
Positive SVS	75/333 (22.5)	53/380 (13.9)	0.67 (0.41–1.10)	
SICH^a^				.73
Negative SVS	5/83 (6.0)	5/43 (11.6)	0.91 (0.17–4.76)	
Positive SVS	21/307 (6.8)	35/343 (10.2)	1.58 (0.83–3.01)	
FPE				.67
Negative SVS	29/80 (36.2)	18/44 (40.9)	1.05 (0.43–2.58)	
Positive SVS	125/323 (38.7)	151/369 (40.9)	0.98 (0.70–1.35)	
Final eTICI 2b-3				.03
Negative SVS	75/87 (86.2)	41/50 (82.0)	0.60 (0.16–1.97)	
Positive SVS	303/354 (85.6)	367/399 (92.0)	2.00 (1.28–3.52)	

^a^Data were missing in 133 patients.

Abbreviations: Adj = adjusted; eTICI = expanded treatment in cerebral infarction; EVT = endovascular thrombectomy; CI = confidence interval; d = days; EVT = endovascular thrombectomy; FPE = first-pass effect (eTICI 2c-3 after the first pass); IQR = interquartile range; IVT = intravenous thrombolysis; mRS = modified Rankin Scale; OR = odds ratio; SVS = susceptibility vessel sign; SWI = susceptibility weighted imaging; SICH = symptomatic intracranial haemorrhage.

Susceptibility vessel sign status was not significantly associated with any of the angiographic outcomes (Table 2). There was an interaction between SVS and IVT for final successful reperfusion (adjusted *P*_interaction_ = .03); that is, IVT plus EVT was associated with higher odds of successful reperfusion in patients with SVS (aOR = 2.00; 95% CI, 1.28–3.52) compared to EVT alone, whereas there was no association between IVT and final reperfusion in patients without SVS (aOR = 0.60; 95% CI, 0.16–1.97); see Table 3.

### Secondary analyses using 3-class SVS as a categorical variable

There were some baseline imbalances between the 3 classes of SVS. Patients with SVS++ were younger and more severe clinically, with larger strokes and proximal occlusions and more frequently received IVT compared to patients with SVS+. Baseline characteristics by 3-class SVS status are summarised in Table S4.

When assessing the 3-class SVS variable, SVS++ and not SVS+ was associated with 90-day mRS 0–2 and ordinal mRS in the adjusted analyses. Both SVS++ and SVS+ were associated with lower 90-day mortality, but not with SICH (Table S5). No interaction was found between 3-class SVS and IVT for all clinical outcomes (Table S6).

SVS++ was associated with final successful reperfusion in the unadjusted model but not in the adjusted model (aOR = 1.33; 95% CI, 0.72–2.46). No association was seen with the other angiographic outcomes (Table S5). There was an interaction between 3-class SVS and IVT for final successful reperfusion (adjusted *P*_interaction_ = .02). Intravenous thrombolysis was associated with higher odds of successful reperfusion only in the SVS++ subgroup (aOR = 3.2; 95% CI, 1.50–6.77), see Table S6.

### Secondary analyses using 3-class SVS as an ordinal variable

When SVS was modelled as an ordinal variable, each step increase was associated with 90-day mRS 0–2 (aOR 1.61; 95% CI, 1.23–2.10), ordinal mRS (aOR 0.67; 95% CI, 0.54–0.81) and mortality (aOR 0.62; 95% CI, 0.46–0.85), but not with SICH (aOR 1.14; 95% CI, 0.75–1.74) (Table S5). No interaction was found for all clinical outcomes.

Ordinal SVS was not associated with the first-pass effect nor final successful reperfusion (Table S5). There was an interaction between ordinal SVS and IVT for final successful reperfusion (adjusted *P*_interaction_ = .03) but not with first-pass effect (Table S6).

## Discussion

In this analysis from the ETIS registry, we included 909 patients who underwent EVT of anterior circulation vessel occlusion and investigated the potential interaction between SVS and IV thrombolysis in patients with AIS. Our main findings are the following: (1) SVS was associated with clinical outcomes in acute stroke patients whether IVT is given or not before EVT, (2) SVS did not significantly affect angiographic outcomes; however, IVT before EVT improved the rate of final successful reperfusion in patients with SVS compared to EVT alone and (3) in patients without SVS, IVT plus EVT did not impact the rate of successful reperfusion compared to EVT alone.

Our study confirmed that the presence of SVS was significantly associated with improved clinical outcomes, as measured by the mRS at 90 days. This association remained significant after adjusting for potential confounders, indicating that SVS could be an important imaging marker for predicting favourable recovery in AIS patients treated with EVT. This finding is in line with other observations.^10^^,^^11^ In addition, only SVS++ (thrombus hyposignal larger than the contralateral artery) was associated with better functional outcomes and lower mortality, whereas SVS+ was not. The mechanisms underlying this positive correlation between SVS and clinical outcomes might be complex and multifactorial, and not completely explained by successful reperfusion as was shown in this study and previous reports.^10^^,^^12^ Thrombi that are SVS− are associated with a high fibrin and platelet content and seem to present a stiffer and more elastic structure^13^ as well as a significantly higher coefficient of friction,^14^ which makes them more difficult to retrieve and often requires multiple thrombectomy attempts, all of which are correlated with poor prognosis.^2^^,^^15^ Another possible factor to consider could be that, as SVS has been linked to cardio embolism^8^: patients whose stroke was caused by cardioembolic diseases might respond better to anticoagulant therapy and suffer from less comorbidities than patients with severe atherosclerosis, thus leading to better clinical outcomes after stroke treatment, as was suggested by the work of Yang et al.^16^ However, in our study, stroke aetiology did not significantly differ between SVS− and SVS+ groups, even though the proportion of cardioembolic strokes was non-significantly higher in the SVS+ group. This suggests that stroke aetiology might only be one of many factors to influence the presence of SVS.

The key objective of this study was to explore the interaction between SVS and IVT in stroke patients treated with EVT. While SVS was associated with favourable clinical outcomes, the effect of IVT was not modified by SVS status for clinical outcomes at 90 days. This may suggest that, although SVS is a positive prognostic indicator, it does not modify the effect of IVT significantly in a direct manner. However, when examining reperfusion outcomes, treatment by IVT plus EVT in SVS patients was associated with significantly higher odds of achieving successful reperfusion compared to EVT alone in SVS patients. This effect was even more pronounced in thrombi with larger hyposignals on MRI, suggesting its stronger correlation with increased red blood cell content within the thrombus.^7^ Although SVS modified the effect of IVT on final eTICI, this did not translate into a significant interaction for functional outcome. Clinical recovery after a stroke is influenced by many additional factors beyond reperfusion such as infarct volume and complications. Also, EVT itself is highly effective, which may attenuate any additional benefit of IVT. Moreover, detecting effect modification on functional outcome typically requires larger sample sizes than for angiographic endpoints.

This finding is consistent with the hypothesis suggested by the work of Vandelanotte et al.,^2^ according to which red blood cell-rich thrombi are more sensitive to thrombolysis. A possible, yet not well-studied, explanation would be that red blood cell-rich thrombi, being less dense in fibrin, provide thrombolytic agents easier access to fibrin mesh, allowing them to partially disintegrate the thrombus, making it simpler to be removed by thrombectomy manoeuvres. However, given the few published works available on the matter, further research is warranted to corroborate this theory. Nevertheless, in our study, the interaction observed between SVS and IVT on final eTICI scores suggests that the presence of SVS, particularly with larger hyposignal (SVS++), could enhance the response to thrombolytic therapy by improving the potential for successful reperfusion in patients undergoing EVT.

Although we found this positive interaction for final reperfusion, we did not find a significant one for any clinical outcome, and that may be due to several elements. Clinical outcomes constitute multifactorial endpoints; though reperfusion may play a role, it is far from being the only factor. It is possible that our study was not statistically powerful enough to demonstrate this relation. Indeed, EVT already being a very effective treatment in itself, the benefit gained from additional thrombolysis on SVS thrombi may have been too small to be demonstrated by our study. Another possible explanation could be that clinical outcomes were assessed at 3 months, and studies have shown that even though the major part of recovery usually happens before 3 months, progress can still be made up to 6 months and beyond.^17^^,^^18^

The *post hoc* analysis of the SWIFT DIRECT trial by Beyeler et al.^19^ had a similar approach to our study. They reported better pre-interventional partial reperfusion rates when IVT was administered before EVT in SVS+ patients. It is worth noting that their study had fewer patients than our study (197 vs 909). Their work also highlights another point similar to our study, which is the imbalance between SVS+ and SVS− groups. Indeed, in our study, SVS+ patients represented 84.5% of all patients and 92% in their study. This high prevalence could have been explained by the time-dependent visibility of SVS shown in a previous study, in which the authors found that the prevalence of SVS increases with time, reaching around 70% at 3 h from symptom onset and 80% at 6 h from symptom onset.^20^ However, in our study, there were no significant differences in time from onset to arterial puncture between SVS− and SVS+ groups, which is likely a good indicator of time from onset to first MRI imaging, meaning that our work does not reinforce the theory of time-dependent visibility of SVS. Another interesting finding in our study is the difference in prevalence of SVS according to the MRI sequence (T2^*^-GRE or SWI). Indeed, SVS prevalence was 75% for the T2^*^-GRE sequence and 91% for the SWI sequence. This result hints at a possible higher sensitivity of the SWI sequence for detecting SVS in AIS, also shown in a prior study.^21^

In a previous work by Zhou et al.,^22^ the interaction between alteplase and hyperdense artery sign (HAS) was assessed on CT imaging in AIS. SVS and HAS seem to correlate well,^23^ SVS being probably slightly more sensitive and slightly less specific. They found a significant positive interaction between HAS and IV alteplase on clinical outcomes and final successful reperfusion, a relation that we failed to demonstrate in our present study. One of the possible reasons might be the lower specificity of SVS compared to HAS. Susceptibility vessel sign can also be seen in calcified thrombi or occlusions due to intracranial atherosclerotic stenosis which may have offset the expected higher rate of successful reperfusion.

We acknowledge several limitations. First, its retrospective nature means that causality cannot be firmly established, and the potential for unmeasured confounding factors remains. However, to our knowledge, this is the largest study investigating the interaction of SVS with IVT. Second, our study was conducted on a random sample of the ETIS registry rather than the entire cohort; thus, some degree of selection bias cannot be fully excluded, although sampling was performed randomly. Third, although we adjusted for a variety of covariates, our study did not explore the exact mechanisms by which SVS influences reperfusion and treatment response as thrombus histology was not available for this study. Fourth, we could not restrict our cohort to patients within the 4.5-h window, as onset-to-imaging data were not available. This may have introduced heterogeneity in IVT eligibility. To reduce this bias, we adjusted analyses for onset-to-arterial access time and other key confounders. Fifth, our analyses did not adjust for potential inter-centre differences in practices. Nevertheless, we adjusted for key patient and procedural characteristics, which reduces but does not completely eliminate this concern. Sixth, other thrombus features on MRI, such as SVS length and volume, were not assessed, so we were not able to include them in this study. However, we also used a visual assessment of the size of SVS with a good interrater agreement to differentiate between types of thrombi, which is simpler and faster than the overestimation ratio of SVS used in previous studies.^24^ We could not assess patients with early recanalisation after IVT, as first angiographic run data were not available. However, prior trials and registries suggest that early recanalisation occurs in less than a third of patients receiving IVT.^25^^,^^26^ Our study would therefore have been underpowered to detect an interaction between SVS and IVT for this outcome. Finally, our results mainly apply to patients treated with IV alteplase. As IV tenecteplase is non-inferior to IV alteplase and many centres have switched to tenecteplase, further research is needed before generalising our results to patients treated with tenecteplase.^27^

## Conclusion

In this substudy of a multicentre prospective EVT registry, we found that the presence of SVS was associated with clinical outcomes regardless of the administration of IVT. The presence of SVS impacted positively the effect of IVT on achieving final successful reperfusion. Further research is necessary to clarify the mechanisms underlying the interaction between SVS and IVT and to validate the clinical utility of SVS in guiding stroke treatment decisions.

## Supplementary Material

aakaf003_Supplement_updated
