# Supplementary material for: Does the susceptibility vessel sign influence the effectiveness of intravenous thrombolysis before endovascular thrombectomy in acute ischaemic stroke?
Source: Eur Stroke J. 2026 Jan 1;11(1):aakaf003. doi: 10.1093/esj/aakaf003 (PMC12866273; doi:10.1093/esj/aakaf003)
Supplement: aakaf003_Supplement_updated [file aakaf003_supplement_updated.docx]

**Supplemental Material**

**Does the susceptibility vessel sign influence the effectiveness of intravenous thrombolysis before endovascular thrombectomy in acute ischemic stroke?**

**Supplemental Table 1. Comparison of baseline characteristics between included and excluded patients**

|  | Included (n=909) | Excluded (n=2492) | P value |
| --- | --- | --- | --- |
| Age, median (IQR) | 73 (60-82) | 73 (61 – 81) | 0.74 |
| Female sex | 462 (50.8) | 1191 (47.8) | 0.53 |
| Medical history |  |  |  |
| Hypertension | 501 (56.6) | 1487 (59.7) | 0.09 |
| Diabetes | 136 (15.5) | 448 (18.0) | 0.09 |
| Dyslipidemia | 261 (29.8) | 805 (32.3) | 0.20 |
| Atrial fibrillation | 172 (19.8) | 448 (18.0) | 0.26 |
| Previous stroke | 116 (13.1) | 349 (14.1) | 0.49 |
| Prestroke mRS 0-2 | 794 (94.5) | 2317 (93.1) | 0.49 |
| Smoking | 137 (16.4) | 523 (21.4) | **0.002** |
| Antithrombotic medication | 333 (38.1) | 922 (37.1) | 0.54 |
| IV thrombolysis | 461 (50.7) | 1295 (53.0) | 0.24 |
| Baseline NIHSS, median (IQR) | 16 (10 - 20) | 15 (9 – 19) | **<0.001** |
| Onset to arterial access time | 255 (188 - 335) | 276 (209 – 368) | **<0.001** |
| Stroke etiology |  |  | 0.08 |
| Atherosclerosis | 118 (13.9) | 388 (15.6) |  |
| Cardioembolic | 348 (41.1) | 1069 (42.9) |  |
| Other | 381 (45.0) | 1034 (41.5) |  |
| Imaging characteristics |  |  |  |
| Occlusion location |  |  | **<0.01** |
| ICA | 208 (23.1) | 523 (21.1) |  |
| M1-MCA | 516 (57.3) | 1191 (47.8) |  |
| M2-MCA | 141 (15.6) | 371 (14.9) |  |
| Vertebro-basilar | 0 (0) | 237 (9.5) |  |
| Other/Distal occlusions | 36 (4.0) | 157 (6.3) |  |
| Baseline ASPECTS (IQR) | 8 (6-9) | 8 (6 – 9) | 0.55 |

**Supplemental Table 2. Comparison of baseline characteristics between IVT+EVT and EVT subgroups**

|  | EVT (n=448) | IVT+EVT (n=461) | P value |
| --- | --- | --- | --- |
| Age, median (IQR) | 72 (60-81) | 74 (61 – 83) | 0.18 |
| Female sex | 231 (51.6) | 231 (50.1) | 0.69 |
| Medical history |  |  |  |
| Hypertension | 252 (57.9) | 249 (55.3) | 0.45 |
| Diabetes | 71 (16.5) | 65 (14.5) | 0.46 |
| Dyslipidemia | 122 (28.6) | 139 (31.1) | 0.42 |
| Atrial fibrillation | 114 (26.9) | 58 (13.1) | **<0.01** |
| Previous stroke | 68 (15.6) | 48 (10.7) | **0.04** |
| Smoking | 77 (18.7) | 60 (14.1) | 0.09 |
| Antithrombotic medication | 196 (45.5) | 137 (31.0) | **<0.01** |
| Prestroke mRS 0-2 | 389 (94.2) | 404 (94.9) | 0.72 |
| Baseline NIHSS, median (IQR) | 16 (10 - 20) | 16 (10 – 20) | 0.76 |
| Onset to arterial access time | 258 (180 - 357) | 252 (191 – 310) | **0.04** |
| Stroke etiology |  |  | 0.62 |
| Atherosclerosis | 63 (15.1) | 54 (12.8) |  |
| Cardioembolic | 171 (40.9) | 177 (41.2) |  |
| Other | 184 (44.0) | 197 (45.9) |  |
| Imaging characteristics |  |  |  |
| MRI sequence |  |  | 0.39 |
| T2*-GRE | 239 (50.7) | 232 (49.3) |  |
| SWI | 209 (47.7) | 229 (52.3) |  |
| Occlusion location |  |  | 0.96 |
| ICA | 101 (22.6) | 107 (23.6) |  |
| M1-MCA | 257 (57.5) | 259 (57.0) |  |
| M2-MCA | 72 (16.1) | 69 (15.2) |  |
| Other/Distal occlusions | 17 (3.8) | 19 (4.2) |  |
| Baseline ASPECTS (IQR) | 8 (6-9) | 8 (6 – 9) | 0.28 |

**Supplemental Table 3. Comparison of baseline characteristics between SWI and T2*-GRE subgroups**

|  | SWI (n=471) | T2*-GRE (n=438) | P value |
| --- | --- | --- | --- |
| Age, median (IQR) | 72 (62-82) | 73 (60 – 82) | 0.99 |
| Female sex | 227 (48.2) | 220 (50.2) | 0.55 |
| Medical history |  |  |  |
| Hypertension | 250 (53.7) | 251 (59.7) | 0.08 |
| Diabetes | 67 (14.5) | 69 (16.5) | 0.45 |
| Dyslipidemia | 110 (23.9) | 151 (36.4) | **<0.001** |
| Atrial fibrillation | 83 (18.3) | 89 (21.5) | 0.23 |
| Previous stroke | 51 (11.0) | 65 (15.5) | 0.06 |
| Smoking | 65 (14.5) | 72 (18.5) | 0.13 |
| Antithrombotic medication | 165 (35.9) | 168 (40.5) | 0.16 |
| Prestroke mRS 0-2 | 410 (95.3) | 383 (93.6) | 0.29 |
| Baseline NIHSS, median (IQR) | 15 (10 - 19) | 16 (11 – 20) | 0.17 |
| Onset to arterial access time | 233 (182 - 315) | 278 (193 – 354) | **<0.001** |
| Stroke etiology |  |  | **0.02** |
| Atherosclerosis | 49 (11.1) | 69 (17.0) |  |
| Cardioembolic | 179 (40.6) | 169 (41.6) |  |
| Other | 213 (48.3) | 168 (41.4) |  |
| Imaging characteristics |  |  |  |
| Occlusion location |  |  | **<0.001** |
| ICA | 90 (19.4) | 118 (26.9) |  |
| M1-MCA | 261 (57.3) | 255 (58.2) |  |
| M2-MCA | 85 (18.4) | 56 (12.8) |  |
| Other/Distal occlusions | 27 (5.8) | 9 (2.1) |  |
| Baseline ASPECTS (IQR) | 7 (6-9) | 8 (7 – 10) | **0.002** |

GRE: T2*-weighted gradient echo, SWI: susceptibility-weighted imaging

**Supplementary Table 4. Baseline characteristics stratified by three-class SVS status (N=909).**

|  | SVS - (n=141) | SVS + (n=339) | SVS++ (n=429) | P value |
| --- | --- | --- | --- | --- |
| Age, median (IQR) | 68 (57-80) | 75 (64 – 84) | 72 (60 – 80) | **0.01** |
| Female sex | 79 (56.0) | 175 (51.6) | 208 (48.5) | 0.28 |
| Medical history |  |  |  |  |
| Hypertension | 72 (54.1) | 207 (61.8) | 222 (53.2) | 0.05 |
| Diabetes | 27 (20.6) | 57 (17.1) | 52 (12.6) | 0.05 |
| Dyslipidemia | 41 (31.3) | 92 (28.1) | 108 (30.8) | 0.65 |
| Atrial fibrillation | 21 (15.8) | 72 (22.0) | 79 (19.4) | 0.31 |
| Previous stroke | 22 (16.4) | 45 (13.5) | 49 (11.8) | 0.37 |
| Smoking | 27 (20.8) | 49 (15.6) | 61 (15.6) | 0.34 |
| Antithrombotic medication | 55 (40.4) | 125 (38.7) | 153 (37.0) | 0.73 |
| IV thrombolysis | 51 (36.2) | 175 (51.6) | 235 (54.8) | **<0.001** |
| Baseline NIHSS, median (IQR) | 15 (9 - 19) | 14.5 (9 – 20) | 17 (12 – 20) | **0.01** |
| Onset to arterial access time | 265 (180 - 351) | 257 (185 – 338) | 252 (192 – 319) | 0.75 |
| Stroke etiology |  |  |  | 0.35 |
| Atherosclerosis | 21 (16.4) | 47 (14.6) | 50 (12.6) |  |
| Cardioembolic | 45 (35.2) | 142 (44) | 161 (40.7) |  |
| Other | 62 (48.4) | 134 (41.4) | 185 (46.7) |  |
| Imaging characteristics |  |  |  |  |
| MRI sequence^#^ |  |  |  | **<0.001** |
| T2*-GRE | 100 (22.8) | 176 (40.2) | 162 (37.0) |  |
| T2*-SWI | 41 (8.7) | 163 (34.6) | 267 (56.7) |  |
| Occlusion location |  |  |  | **0.02** |
| ICA | 27 (19.2) | 64 (19.1) | 117 (27.5) |  |
| M1-MCA | 83 (58.9) | 192 (57.3) | 241 (56.7) |  |
| M2-MCA | 27 (19.2) | 63 (18.8) | 51 (12.0) |  |
| Other/Distal occlusions | 4 (2.8) | 16 (4.8) | 16 (3.8) |  |
| Baseline ASPECTS (IQR) | 8 (5-9) | 8 (6 – 10) | 7 (6 – 9) | **0.02** |

^#^Numbers in parentheses are row-wise proportions

Abbreviations :ASPECTS= Alberta Stroke Program Early CT Score, EVT= Endovascular Thrombectomy, GRE= Gradient Echo, ICA= Internal Carotid Artery, IQR= Interquartile Range, IV= Intra-Venous, MCA= Middle Cerebral Artery, MRI= Magnetic Resonance Imaging, NIHSS= National Institutes of Health Stroke Score, SVS= Susceptibility Vessel Sign, , SWI= Susceptibility Weighted Imaging.

**Supplementary Table 5. Association of categorical and ordinal three-class SVS with clinical and angiographic outcomes**

|  | SVS – (n=141) | SVS+ (n=339 | SVS++ (n=429) | Odds ratio* | Adj OR (95% CI) | Adj OR (95% CI) for ordinal 3-class SVS |
| --- | --- | --- | --- | --- | --- | --- |
| 90d mRS 0-2 | 41 (31.8) | 123 (40.2) | 194 (47.4) | SVS+ | 1.58 (0.89 – 2.80) | 1.61 (1.23–2.10) |
|  |  |  |  | SVS++ | 2.58 (1.45 – 4.58) |  |
| 90d mRS (median [IQR]) | 4 (2-6) | 3 (1-5) | 3 (1-4) | SVS+ | 0.52 (0.42 – 1.01) | 0.67 (0.54–0.81) |
|  |  |  |  | SVS++ | 0.37 (0.27 – 0.67) |  |
| 90d mortality | 35 (27.1) | 155 (18.0) | 73 (17.9) | SVS+ | 0.54 (0.30 – 0.98) | 0.62 (0.46–0.85) |
|  |  |  |  | SVS++ | 0.42 (0.23 – 0.76) |  |
| SICH* | 10 (7.9) | 19 (6.6) | 37 (10.2) | SVS+ | 0.68 (0.27 – 1.66) | 1.14 (0.75–1.74) |
|  |  |  |  | SVS++ | 1.07 (0.46 – 2.49) |  |
| FPE | 47 (37.9) | 119 (39.4) | 157 (40.3) | SVS+ | 0.98 (0.66 – 1.60) | 0.98 (0.78–1.22) |
|  |  |  |  | SVS++ | 0.96 (0.68 – 1.65) |  |
| Final eTICI 2b-3 | 116 (84.7) | 288 (86.7) | 382 (90.7) | SVS+ | 1.03 (0.57 – 1.89) | 1.23 (0.89–1.70) |
|  |  |  |  | SVS++ | 1.33 (0.72 – 2.46) |  |

*Negative represent the reference category and is not shown.

Abbreviations :

Adj= Adjusted, CI= Confidence Interval, d= days, eTICI= expanded Treatment in Cerebral Infarction, mRS= modified Rankin Scale, FPE= First Pass Effect (eTICI 2c-3 after the first pass), OR= Odds Ratio, SVS= Susceptibility Vessel Sign, Unadj= Unadjusted. SICH= symptomatic intracranial hemorrhage.

*data were missing in 133 patients.

**Supplemental Table 6. Treatment effect of combined IVT and EVT versus EVT alone for the study outcomes stratified by three-class categorical and ordinal SVS status.**

|  | EVT alone (n/N [%]) | IVT+EVT (n/N [%]) | Adj OR (95% CI) | P interaction for categorical 3-class SVS | P interaction for ordinal 3-class SVS |
| --- | --- | --- | --- | --- | --- |
| 90d mRS 0-2 |  |  |  |  |  |
| Negative SVS | 21/83 [25] | 20/46 [43] | 3.52 (0.98-12.54) | Reference | 0.19 |
| Positive SVS (+) | 52/151 [34] | 71/155 [46] | 1.80 (0.94-3.43) | 0.57 |  |
| Positive SVS (++) | 74/182 [41] | 120/225 [53] | 1.20 (0.71-2.01) | 0.27 |  |
| 90d mRS *(median [IQR])* |  |  |  |  |  |
| Negative SVS | 4 [4] | 3.5 [5] | 0.56 (0.32-1.38) | Reference | 0.44 |
| Positive SVS (+) | 3 [3] | 3 [3] | 0.67 (0.42-1.07) | 0.72 |  |
| Positive SVS (++) | 3 [4] | 2 [3] | 0.89 (0.59-1.35) | 0.83 |  |
| 90d mortality |  |  |  |  |  |
| Negative SVS | 21/83 [25] | 14/46 [30] | 0.98 (0.45-3.10) | Reference | 0.99 |
| Positive SVS (+) | 35/151 [23] | 20/155 [13] | 0.50 (0.20-0.83) | 0.09 |  |
| Positive SVS (++) | 40/182 [22] | 33/225 [15] | 0.89 (0.39-1.36) | 0.37 |  |
| SICH* |  |  |  |  |  |
| Negative SVS | 5/83 [6] | 5/43 [11] | 0.91 (0.17-4.76) | Reference | 0.81 |
| Positive SVS (+) | 9/143 [6] | 10/146 [7] | 1.28 (0.43-3.75) | 0.56 |  |
| Positive SVS (++) | 12/164 [7] | 25/197 [12] | 1.77 (0.77-4.05) | 0.89 |  |
| FPE |  |  |  |  |  |
| Negative SVS | 29/80 [36] | 18/44 [41] | 1.05 (0.43-2.58) | Reference | 0.15 |
| Positive SVS (+) | 51/148 [34] | 68/154 [44] | 1.41 (0.93-2.40) | 0.58 |  |
| Positive SVS (++) | 74/175 [42] | 83/215 [39] | 0.69 (0.52-1.20) | 0.40 |  |
| Final eTICI 2b-3 |  |  |  |  |  |
| Negative SVS | 75/87 [86] | 41/50 [82] | 0.62 (0.22-1.72) | Reference | **0.03** |
| Positive SVS (+) | 138/162 [85] | 150/170 [88] | 1.38 (0.70-2.90) | 0.12 |  |
| Positive SVS (++) | 165/192 [86] | 217/229 [95] | 3.20 (1.50-6.77) | 0.02 |  |

Abbreviations :

Adj= Adjusted, eTICI= expanded Treatment in Cerebral Infarction, EVT= Endovascular Thrombectomy, CI= Confidence Interval, d= days, EVT= Endovascular Thrombectomy, FPE= First Pass Effect (eTICI 2c-3 after the first pass), IQR= Interquartile Range, IVT= Intra-Venous Thrombolysis, mRS= modified Rankin Scale, OR= Odds Ratio, SVS= Susceptibility Vessel Sign, SWI= Susceptibility Weighted Imaging. SICH= symptomatic intracranial hemorrhage.

*data were missing in 133 patients.
